# Data on validation using accuracy profile of HPLC-UV method

**DOI:** 10.1016/j.dib.2019.103877

**Published:** 2019-03-26

**Authors:** Ahmed M. Ibrahim, Hassan A.M. Hendawy, Wafaa S. Hassan, Abdalla Shalaby, Heba M. El-sayed

**Affiliations:** a National Organization for Drug Control and Research, P.O. Box 29, Cairo, Egypt; b Department of Analytical Chemistry, Faculty of Pharmacy, Zagazig University, Zagazig, Egypt

**Keywords:** Accuracy profile, HPLC, Ascorbic acid, Paracetamol, Guaifenesin

## Abstract

The data presented are related to the article entitled “Six Sigma quality approach for HPLC-UV method optimization” Ibrahim et al., 2019. The raw data of HPLC analysis of ascorbic acid (AS), paracetamol (PA) and guaifenesin (GU) are presented. Calibration standards were prepared at six concentrations levels (25%, 50%, 75%, 100%, 125% and 150%) each day and measured in triplicate. Validation standards were prepared at four concentration levels (25%, 60%, 100% and 150%) each day and measured in quintet. Three different series were used for method validation and prepared at the rate of one series per day.

**Table:** Specifications table

Subject area	*Chemistry, Chromatography, Pharmaceutical science*
More specific subject area	*Validation*
Type of data	*Table and figure*
How data was acquired	*The analyses were performed using A Dionex UltiMate 3000 HPLC system equipped with a MWD−30,000 (RS) detector, a TCC-3×00(RS) column compartment oven, a LPG-3400SD Quaternary pump, and remote injector with Chromeleon®7 software.*
Data format	*Raw*
Experimental factors	*Ascorbic acid (AS), Paracetamol (PA) and Guaifenesin (GU). Stock solutions were prepared in methanol containing 2.5, 3.25, 1.*0 mg ml^−1^*of AS, PA and GU, respectively. Calibration standards were prepared by dilution of the stock standard solutions with buffer. The validation standards were prepared taking into considerations the effervescent granules matrix.*
Experimental features	*Three replicate for six concentration level were repeated for three days (calibration set). Five replicate for four concentration level were repeated for three days (validation set).*
Data source location	*Cairo, Egypt.*
Data accessibility	*The Data are available with this article*
Related research article	*“Six Sigma quality approach for HPLC-UV method optimization”* *Ahmed M. Ibrahim, Hassan A.M. Hendawy, Wafaa S. Hassan, Abdalla Shalaby, Heba M. El-sayed. Microchemical Journal**[1]**.*

**Table:** 

**Value of the data** • The use of validated methods is important for an analytical laboratory to show its qualification and competency Table 1, Table 2. **Table 1:** Calibration set. Concentration level First day Second day Third day AS(mAU) PA(mAU) GU(mAU) AS(mAU) PA(mAU) GU(mAU) AS(mAU) PA(mAU) GU(mAU) 25% 10.689 56.742 14.456 10.636 56.969 14.414 10.665 56.877 14.39 10.681 54.103 14.003 10.531 52.972 13.963 10.377 52.886 14.032 10.677 54.263 14.024 10.666 54.324 13.866 10.675 53.983 13.907 50% 21.156 101.945 27.198 21.198 101.116 27.311 21.078 101.349 27.099 21.15 101.99 27.245 21.15 102.628 27.101 21.243 102.327 27.246 21.128 101.932 27.288 21.065 101.602 27.062 21.017 101.135 27.146 75% 32.535 151.523 41.696 32.372 155.903 42.132 33.027 156.809 42.42 32.628 151.676 41.797 32.432 151.373 41.834 32.387 151.447 41.923 32.571 151.791 41.848 32.343 154.17 42.376 32.924 155.249 42.488 100% 42.494 192.624 54.309 41.857 189.219 54.016 41.339 188.653 53.989 42.51 192.876 54.372 41.957 191.461 54.308 41.742 191.335 54.083 42.486 192.781 54.394 42.274 191.838 54.435 42.129 191.917 54.34 125% 53.399 234.045 67.952 53.132 235.233 69.676 53.321 238.548 69.142 52.94 233.698 67.907 53.205 232.91 67.652 53.079 232.425 67.476 52.949 234.122 67.947 52.525 232.999 68.829 52.349 234.679 68.994 150% 63.259 268.373 80.922 63.069 270.466 80.738 63.414 270.124 80.985 63.221 267.938 80.883 62.652 266.519 81.025 62.42 266.778 81.559 63.244 268.168 80.762 63.56 273.176 81.418 64.391 274.409 80.635 **Table 2:** Validation set. Concentration level First day Second day Third day AS(mAU) PA(mAU) GU(mAU) AS(mAU) PA(mAU) GU(mAU) AS(mAU) PA(mAU) GU(mAU) 25% 10.807 55.015 14.218 10.776 54.740 14.275 10.722 54.959 14.233 10.902 55.505 14.308 10.870 54.728 14.009 10.718 53.583 13.968 10.764 54.816 14.148 10.642 54.761 14.164 10.632 54.822 14.004 10.714 54.726 14.164 10.758 54.835 14.049 10.780 54.390 14.107 10.633 54.457 14.088 10.577 54.457 14.176 10.577 54.798 14.101 60% 25.493 121.059 32.709 25.282 120.696 32.603 25.206 120.305 32.333 25.473 121.107 32.727 25.740 120.501 33.673 25.611 123.985 34.025 25.486 121.014 32.73 25.509 120.288 32.665 25.356 120.047 32.693 25.448 121.068 32.866 25.769 120.221 33.381 25.588 122.105 33.802 25.547 121.517 32.878 25.409 119.694 32.297 25.028 117.579 32.123 100% 42.645 193.349 54.537 42.595 190.835 54.137 42.041 189.436 54.073 42.591 193.309 54.506 42.623 192.342 54.239 42.410 191.401 54.280 42.582 193.325 54.503 43.662 192.358 54.780 43.444 193.335 56.169 42.562 193.196 54.612 42.402 194.162 54.428 42.614 193.507 54.224 42.537 193.156 54.557 43.089 191.611 54.295 42.744 190.692 55.000 150% 63.282 268.914 81.076 63.138 268.107 81.708 62.949 270.198 81.522 63.4 268.842 81.077 63.511 266.422 80.647 62.940 265.011 80.789 63.261 269.486 80.973 63.775 270.833 82.485 64.094 275.891 83.155 63.31 269.579 80.962 63.437 272.275 80.557 64.071 270.913 80.718 63.293 268.708 80.929 63.166 267.364 81.334 62.851 268.701 81.171 • The validation of quantitative analytical procedures is a great challenge for the quality control of industrial chemicals, specially concerning to the statistical parameters for accuracy criteria. • The present data shows relevant information that complement the related article and can be interesting for readers of the Data in Brief. • The researchers can compare different regression models other than those used in the related article

## 1 Data

The choice of number of calibration standards and validation standards depends on the selected protocol. V2 was the type of the modified protocol.

Calibration standards were prepared at six concentrations levels (25%, 50%, 75%, 100%, 125% and 150%) each day and measured in triplicate.

Validation standards were prepared at four concentration levels (25%, 60%, 100% and 150%) each day and measured in quintet.

Three different series were used for method validation and prepared at the rate of one series per day.

**Fig. 1:**
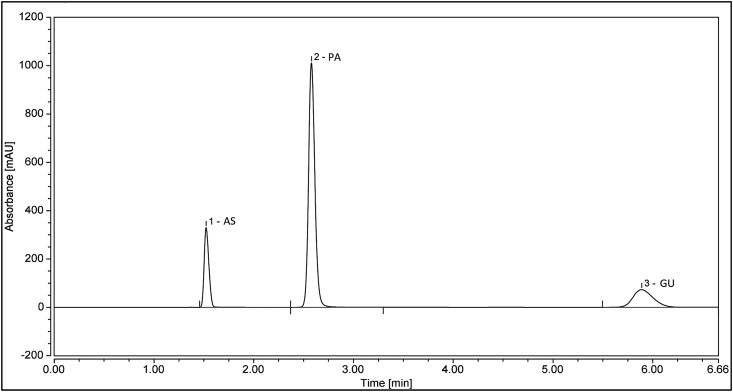
HPLC chromatograms of a validation standard solution containing 0.25 mg ml^−1^ of AS, 0.325 mg ml^−1^ of PA, and 0.10 mg ml^−1^ of GU at optimal condition.

Fig. 1 shows HPLC chromatogram of a validation standard solution containing 0.25 mg ml^−1^ of AS, 0.325 mg ml^−1^ of PA, and 0.10 mg ml^−1^ of GU at optimal condition.

## 2 Experimental design, materials, and methods

AS, PA and GU were supplied by NODCAR. Methanol (HPLC grade), potassium dihydrogen phosphate, and ortho-phosphoric acid 85% were purchased from Merck. A validation matrix solution was prepared by dissolving sodium bicarbonate, citric acid anhydrous, tartaric acid anhydrous, povidone K25, aspartam, and disodium edetate in methanol in order to obtain 24.2, 9.6, 12.3, 1.2, 0.7 and 03 mg ml^−1^, respectively.

The separation was performed on A Phenomenex Luna^®^ CN column, (150 mm × 4.6 mm, 5 μm), using an isocratic mode with a mixture of 50 mM potassium dihydrogen phosphate buffer adjusted with ortho-phosphoric acid to pH 2.2 as the aqueous component of the mobile phase and methanol in the ratio 85.9:14.1 (v/v) at 1 ml/min flow rate, with UV detector at 275 nm and a column temperature of 45 °C. The injected volume was 20 μL.
